# NUAK1 (ARK5) Is Associated with Poor Prognosis in Ovarian Cancer

**DOI:** 10.3389/fonc.2016.00213

**Published:** 2016-10-27

**Authors:** Neil T. Phippen, Nicholas W. Bateman, Guisong Wang, Kelly A. Conrads, Wei Ao, Pang-ning Teng, Tracy A. Litzi, Julie Oliver, G. Larry Maxwell, Chad A. Hamilton, Kathleen M. Darcy, Thomas P. Conrads

**Affiliations:** ^1^National Capital Consortium Fellowship in Gynecologic Oncology, Walter Reed National Military Medical Center, Bethesda, MD, USA; ^2^Department of Defense Gynecologic Cancer Center of Excellence, Women’s Health Integrated Research Center at Inova Health System, Annandale, VA, USA; ^3^Department of Obstetrics and Gynecology, Uniformed Services University of the Health Sciences, Bethesda, MD, USA; ^4^The John P. Murtha Cancer Center, Walter Reed National Military Medical Center, Bethesda, MD, USA; ^5^Department of Obstetrics and Gynecology, Inova Fairfax Medical Campus, Falls Church, VA, USA; ^6^Inova Center for Personalized Health, Inova Fairfax Hospital, Falls Church, VA, USA

**Keywords:** ovarian cancer, NUAK1, ARK5, gene expression, survival, prognosis, migration

## Abstract

**Background and objective:**

Nua kinase 1 (NUAK1) was identified in multigene signatures of survival and suboptimal debulking in high-grade serous ovarian cancer (HGSOC). This study investigates the individual clinical and biologic contributions of NUAK1 in HGSOC patients and cell lines.

**Methods:**

Public transcript expression, clinical, and outcome data were used to interrogate the relationship between NUAK1 and clinicopathologic factors and patient outcomes including progression-free survival (PFS) and molecular subtypes using logistic and Cox modeling. Analysis of NUAK1 transcript expression was performed in primary tumors from 34 HGSOC patients with < or ≥2 years PFS. The impact of silencing NUAK1 by RNA interference (RNAi) on the migratory potential and chemosensitivity of SOC cells was assessed *in vitro*.

**Results:**

Elevated NUAK1 transcript expression was associated with worse PFS (hazard ratio = 1.134), advanced stage (odds ratio, OR = 1.7), any residual disease (OR = 1.58), and mesenchymal disease subtype (OR = 7.79 ± 5.89). Elevated NUAK1 transcript expression was observed in HGSOC patients with < vs. ≥2 years PFS (*p* < 0.045). RNAi-mediated silencing of NUAK1 expression attenuated migration of OV90 and E3 HGSOC cells *in vitro*, but did not modulate sensitivity to cisplatin or paclitaxel.

**Conclusion:**

Elevated NUAK1 was associated with poor survival as well as advanced stage, residual disease after cytoreductive surgery and mesenchymal molecular subtype. NUAK1 impacted migration, but not chemosensitivity, *in vitro*. Additional studies are needed to further develop the concept of NUAK1 as a clinically deployable biomarker and therapeutic target in HGSOC.

## Introduction

High-grade serous ovarian cancer (HGSOC) will comprise approximately 70% of the estimated 21,290 new diagnoses of ovarian malignancy in the US in 2016 (1, 2). Since HGSOC is also responsible for 90% of ovarian cancer cases with peritoneal carcinomatosis, it will be responsible for the vast majority of the 14,180 expected deaths from ovarian cancer in 2016 (1, 2). Though HGSOC is an ominous diagnosis, considerable variation exists between patients in terms of the time from diagnosis to disease progression and cancer death. Some patients progress relatively quickly within the first 1–2 years, while others may take between 5 and 10 years to succumb to their cancer. Common features of shorter progression-free survival (PFS) and overall survival (OS) in HGSOC patients include older age, advanced stage and dissemination of disease into the upper abdominal cavity at diagnosis, and presence of residual tumor following cytoreductive surgery (1, 3, 4). Recently, genomic studies of HGSOC have confirmed the dominant alterations in p53, common defects in homologous recombination, and identified novel gene expression patterns that correlate with OS, PFS, suboptimal debulking, and molecular subtypes (5–8) augmenting clinicopathologic characteristics that inform on HGSOC outcomes and enhancing chemotherapy and surgical planning.

The overexpression of adenosine monophosphate-related kinase 5 (ARK5), also known as novel (nua) kinase family 1 (NUAK1) (9, 10), exemplifies a transcript whose elevated abundance has been identified in several gene expression signatures correlated with inferior survival and suboptimal residual tumor (>1 cm) after cytoreductive surgery in HGSOC (5, 6). The relationship between NUAK1 and either PFS or molecular subtypes in HGSOC has not yet been reported. In addition, the association between NUAK1 and OS in HGSOC patients has not yet been validated. NUAK1 is a downstream effector of Akt and is activated in response to cellular hypoxia and nutrient starvation (10, 11). Activation of NUAK1 leads to enhanced proliferation, invasion, and metastatic potential in lung, colorectal, and pancreatic adenocarcinoma cells (10, 11). There is, however, a paucity of data detailing possible mechanisms responsible for the observed poor survival among HGSOC patients with elevated NUAK1 expression. Recent efforts have described small-molecule inhibitors targeting NUAK1/2 that have demonstrated *in vitro* efficacy against tumor cell proliferation and migratory potential, highlighting the possibility that NUAK1 may represent a therapeutic target (12).

This investigation details the significant associations between elevated NUAK1 transcript expression and poor outcomes in HGSOC, documenting the relationship with PFS and the mesenchymal subtype, and validates previous associations with OS. Elevated NUAK1 transcript expression and shorter PFS was validated by quantitative PCR analyses in ovarian cancer tissue from HGSOC patients with short vs. long disease progression. We further demonstrate that knockdown of NUAK1 does not modulate chemosensitivity, but does confer a pro-migratory phenotype to HGSOC cells *in vitro*. These analyses defined a biologically and clinically compelling combination of biomarkers that when both expressed at high levels indicated worse PFS and OS in HGSOC patients and flagged the molecular subtypes with the worst clinical outcome (mesenchymal and proliferative).

## Materials and Methods

### Biostatistical Analyses of NUAK1 in Public Patient-Derived High Throughput and Clinical Outcome Data

#### Public Dataset Acquisition

Tumor transcript expression data were acquired in the R open source statistical computing and graphing environment version 3.1.2 using the Bioconductor^1^ packages *FULLVcuratedOvarianData_1.0.1* and *curatedOvarianData_1.3.4*., TCGA’s data portals,^2^ the Memorial Sloan Kettering Cancer Center (MSKCC) Cancer Genomics Data Servers using the *CDGS* package in R, and the Gene Expression Omnibus (GEO) repository^3^ (5, 13–15). All downloaded data sets were derived from hybridization-based transcriptomic data generated from fresh frozen primary tumor and were screened for inclusion criteria. Only cases of ovarian tumor from serous histology with complete stage information (I, II, III, IV, early, or late), with available vital status (dead or alive), and survival time in months from the date of diagnosis were included in the analysis (Table 1). Some cases also had PFS data, age at diagnosis, suboptimal disease status, any residual disease status, BRCA1/2 status, and/or up to three distinct molecular subtype classifications. Previously described duplications of data within the data sets were removed, and any perceived duplications within a data set or between data sets from the same collaborations were removed (16).

**Table 1:** **Clinicopathologic characteristics of public transcript datasets analyzed**.

Author accession #	^a^Bonome GSE26712	^a^Denkert GSE14764	^a^Dressman GSE3149	^a^Mok GSE18520	^a^Tothill GSE9891	^a^Mateescu GSE26193	^a^TCGA	^b^Konecny GSE53963	Total
Reference #	(17)	(18)	(19)	(21)	(22)	(20)	(5)	(8)	–
Cases	185	68	116	53	216	79	545	174	1,436
Stage									
I–II	–	3	1	–	29	17	42	8	100
III	149	64	98	53	169	48	421	126	1,128
IV	36	1	17	–	18	14	82	40	208
Age									
<60	70	–	–	–	119	–	288	63	540
≥60	112	–	–	–	97	–	257	111	577
No data	3	68	116	53	–	79	–	–	319
Residual disease									
No gross	90	–	62	53	84	–	115	–	199
<1 cm		–			41	–	244	–	490
≥1 cm	95	–	54	–	65	–	134	–	348
No data	–	68	–	–	26	79	52	174	399
Vital status									
Dead	129	19	67	41	94	60	281	153	844
Alive	56	49	49	12	122	19	264	21	592

*^a^Author name – discovery cohort*.

*^b^Author name – validation cohort*.

#### Statistical Analysis of Expression Data

Eligible cases from seven Affymetrix data sets were combined (GSE26712, GSE14764, GSE3149, GSE18520, GSE9891, GSE26193, and TCGA) (5, 17–22). Prior to any statistical analyses, the seven merged data sets were combat-corrected to correct for any between-experiment batch effects. Batch correction was completed with the *Surrogate Variable Analysis* package from Bioconductor in “R” (15). We further validated our findings correlating elevated NUAK1 and OS in an independent HGSOC cohort derived from cross-microarray platform, i.e., Agilent, data (GSE53963) (8).

Transcript abundance levels of the following probesets in the discovery, Affymetrix cohort (NUAK1_204589_at) and the validation, Agilent cohort (NUAK1_A_23_P348257) were analyzed in univariate Cox logistic regression models for relationship with OS using Bioconductor package *survival_2.37-7* (15). Impact on PFS was similarly calculated in a subset of cases in which time to first disease recurrence information was available. Log-rank analyses with Kaplan–Meier (KM) plots were generated for OS and PFS comparing survival based on low (≤median) vs. high (>median) probe set expression values. All tests were two-sided and significance was set at *p* < 0.05. Odds ratio calculations were performed by logistic regression modeling using the glm function in R statistical computing software (x64 ver 3.2.0) correlating high NUAK1 expression with advanced disease stage, age at diagnosis, having microscopic residual disease, i.e., R0, following cytoreductive surgery, having wild-type BRCA1 and relative to previously published classifications of patients exhibiting mesenchymal-like, differentiated-like, immunoreactive-like, or proliferative-like ovarian cancer molecular disease subtypes (5, 7, 8).

### Quantitative PCR Validation of NUAK1 Expression Levels Relative to Progression-Free Survival

#### Cohort Selection and Laser-Microdissection

De-identified formalin-fixed, paraffin-embedded (FFPE) tissue blocks from patients with advanced-stage HGSOC (*n* = 34) and clinical histories denoting PFS times of ~4.6–77.9 months were obtained from INOVA Fairfax Hospital under an IRB-approved protocol. Eight micrometer thick sections of each tissue were cut by microtome, placed onto PEN membranes (Leica Microsystems, Wetzlar, Germany), and 18.0 mm^2^ of tissue was laser microdissected to selectively capture (> 95% purity) cells of tumor cell populations. Laser-microdissected cells were collected directly into microcentrifuge tubes containing 45.0 μL of digestion buffer, i.e., RecoverAll Total Nucleic Acid Isolation Kit for FFPE (AM1975, Thermo Fisher/Invitrogen).

#### Quantitative PCR Analyses

Total RNA was extracted from equivalent amounts of LMD material using RecoverAll Total Nucleic Acid Isolation Kit for FFPE (AM1975, Thermo Fisher/Invitrogen), and cDNA was prepared from 100 ng of total RNA by reverse transcription using the High Capacity cDNA Reverse Transcription kit (Invitrogen). NUAK1 (Hs00934234_m1) and CDKN1B (Hs00153277_m1) TAQMAN assays were obtained from Invitrogen. cDNA pre-amplification was performed using TAQMAN gene expression assays diluted 1:5 and the TAQMAN PreAmp Master Kit for 10 cycles. Quantitative PCR was performed using TAQMAN gene expression master mix on 10% of total pre-amplified cDNA for 50 cycles (GeneAmp 9700 PCR system, Applied Biosystems). Endpoint data were assembled by comparison of Delta-Ct values for gene of interest vs. corresponding CDKN1B Delta-Ct values, a gene previously reported as a stable pre-amplification and reference control for qPCR analyses of FFPE tissues [Applied Biosystems (23)], for each patient sample. Data reflects triplicate, technical replicate analyses.

### Assessing the Role of NUAK1 in Regulating the Chemosensitivity of OV90 Cells and Cell Migration in Chemosensitive (OV90) and Resistant (E3) Ovarian Cancer Cells

#### Cell Culture and Reagents

OV90 cells were obtained commercially (ATCC, Manassas, VA, USA) and the chemoresistant, HGSOC cell line (E3), previously described (24), was generated in-house from tumor tissues harvested from a patient-derived xenograft mouse established from a chemorefractory ovarian cancer patient. Cell lines were maintained in complete DMEM-F12 media (ATCC) and supplemented with 10% FBS and 1% penicillin/streptomycin (Pen/Strep). Cell line identities were authenticated by STR testing and determined to be mycoplasma free. Cisplatin and paclitaxel were obtained from Sigma-Aldrich.

#### Small Interfering RNA (siRNA) Transfections and Migration Assays

Cell lines were plated in replicates at equivalent densities on day 1, transfected with 125 nM of a scrambled, non-targeting siRNA (ON-TARGETplus Non-targeting Pool, GE Dharmacon, product # D-001810-10-50) or NUAK1-specific siRNA (ON-TARGETplus NUAK1 SMARTpool siRNA, GE Dharmacon, product # L-004931-01-002020) on day 2 and incubated for 48 h. One set of RNA interference (RNAi)-transfected cells were harvested for immunoblot evaluation, while the remaining set was re-plated to achieve confluency 24 h later. Equivalently sized wounds were then generated in confluent cell monolayers and images were collected at identical regions according to the time points specified. Wound healing areas were quantified using cellSens Dimension Software ver. 1.6 (Olympus). Data reflects triplicate measures from biological replicates.

#### Chemotherapy Dose–Response Assay

OV90 cells were transfected with siRNA as described above. However, transfected cells were incubated for 96 h following initial RNAi transfection before being plated equivalently in 96-well plates and incubated overnight. Media was removed and replaced with fresh media containing dose titrations of cisplatin or paclitaxel. Cell viability was assessed 72 h later using the 3-(4,5-dimethylthiazol-2-yl)-5-(3-carboxymethoxyphenyl)-2-(4-sulfophenyl)-2H-tetrazolium (MTS) CellTiter 96 Aqueous One Solution Cell Proliferation Assay (Promega, Madison, WI, USA) according to the manufacturer’s instructions; absorbance (490 nm) was measured using a microplate spectrophotometer (xMark, Bio-Rad) following incubation in MTS reagent at 37°C. Two biological replicates were performed where each cisplatin and paclitaxel dose was assayed in triplicate for each experiment.

#### Immunoblot Analyses

Sub-confluent cells were lysed (1% SDS, 10 mM Tris–HCL pH 7.4), and equivalent amounts of cell lysate were resolved on 4–15% mini-PROTEIN TGX gels and transferred to PVDF membranes. Membranes were blocked for 1 h with 5% non-fat dry milk in 1× TBST and incubated with primary antibody overnight at 4°C. Secondary antibody was incubated for 3 h at ambient temperature followed by incubation in SuperSignal West Dura Chemiluminescent Substrate (ThermoFisher Scientific) for 5 min. Antibodies and concentrations were used as follow: anti-NUAK1 rabbit polyclonal (4458S, Cell Signaling, 1:1000), anti-GAPDH rabbit polyclonal (ab9485, Abcam, 1:1,000), and HRP-linked goat anti-rabbit IgG (Cell Signaling Technologies. 1:1000). Images were acquired using a ChemiDoc XRS+ system (Bio-Rad).

## Results

### Association between Elevated NUAK1 and Worse OS and PFS in Serous Ovarian Cancer Patients

NUAK1 transcript expression was assessed in a cohort of 1,262 patients assembled from seven publicly available, ovarian cancer Affymetrix microarray data sets downloaded from the curatedOvarianData database (14) and an independent cohort of 174 HGSOC patients with Agilent gene expression data (8) (Table 1). Of the ovarian cancer patients with Affymetrix data, 802 also had PFS data. PFS data was not available for the patients with Agilent data. Univariate and multivariate Cox regression modeling revealed that elevated NUAK1 transcript expression was associated with an increased risk of death in both cohorts (Table S1 in Supplementary Material) and disease progression in cohort with PFS data (Table S2 in Supplementary Material).

**Figure 1:**
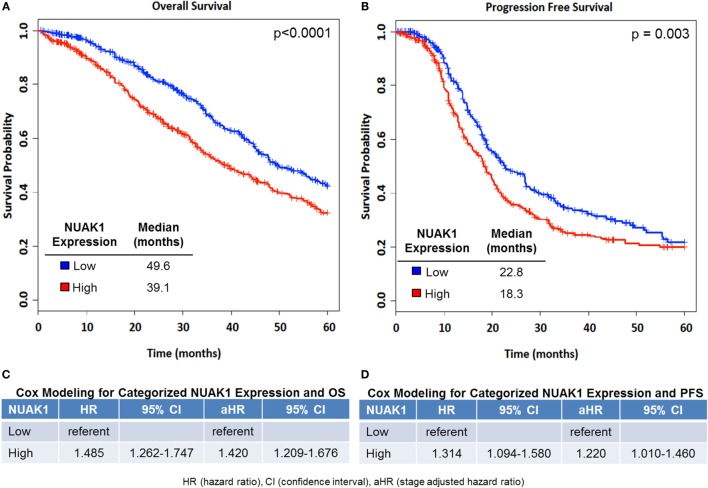
**Elevated NUAK1 expression is associated with poor overall and progression-free survival in high-grade serous ovarian cancer patients**. Kaplan–Meier (KM) plots of NUAK1 transcript expression and cox regression results of categorized NUAK1 expression relative to patient survival. **(A)** Overall survival (OS, NUAK1 continuous multivariate HR = 1.19, *p*-value = 3.03E−07, *n* = 1,262). **(B)** Progression-free survival (PFS, NUAK1 continuous multivariate HR = 1.42, *p*-value = 2.19E−05, *n* = 802). **(C)** Cox regression results of categorized NUAK1 levels versus OS. **(D)** Cox regression results of categorized NUAK1 levels versus PFS. *p*-value insets derived from Log-Rank analyses.

Women with transcript expression of NUAK1 categorized as high vs. low had worse OS (log-rank *p* < 0.0001), with a median survival difference of 10.5 months in high vs. low NUAK1-expressing patients (Figure 1A, Figure S1 in Supplementary Material). Furthermore, elevated NUAK1 transcript expression was associated with poor PFS (*p* = 0.003), with a median survival difference of 4.5 months in patients with high vs. low NUAK1 (Figure 1B). Consistent with these differences in OS and PFS, high vs. low NUAK1 indicated a significant unadjusted and stage-adjusted increased risk of death (Figure 1C) and disease progression (Figure 1D).

**Figure 2:**
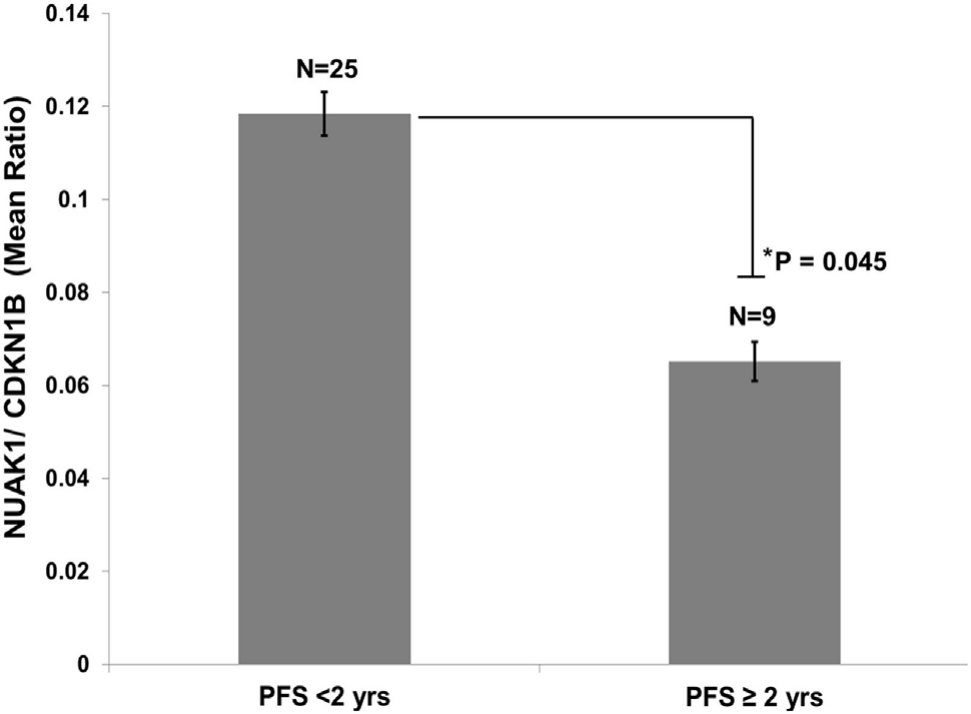
**Elevated NUAK1 transcript expression is associated with poor progression-free survival in an independent cohort of high-grade serous ovarian cancer patients**. Quantitative PCR analyses of formalin-fixed, paraffin-embedded tissues from advanced stage serous ovarian cancer patients exhibiting less than 2 years versus greater than or equal to 2 years progression-free survival (Student’s *t*-test *p* = 0.045). Data reflects triplicate, technical replicate analyses.

Quantitative PCR was performed in FFPE tissues from 25 HGSOC patients with short PFS (<2 years) and 9 HGSOC patients with long PFS (>2 years). All patients were diagnosed with advanced-stage disease. HGSOC patients exhibiting PFS times of less than 2 years expressed higher NUAK1 transcript expression than that in patients who progressed more than 2 years after diagnosis (*p* = 0.045, Figure 2).

### Relationship between Elevated NUAK1 and Older Age, Advanced Stage, Any Residual Disease and Mesenchymal Subtype

**Figure 3:**
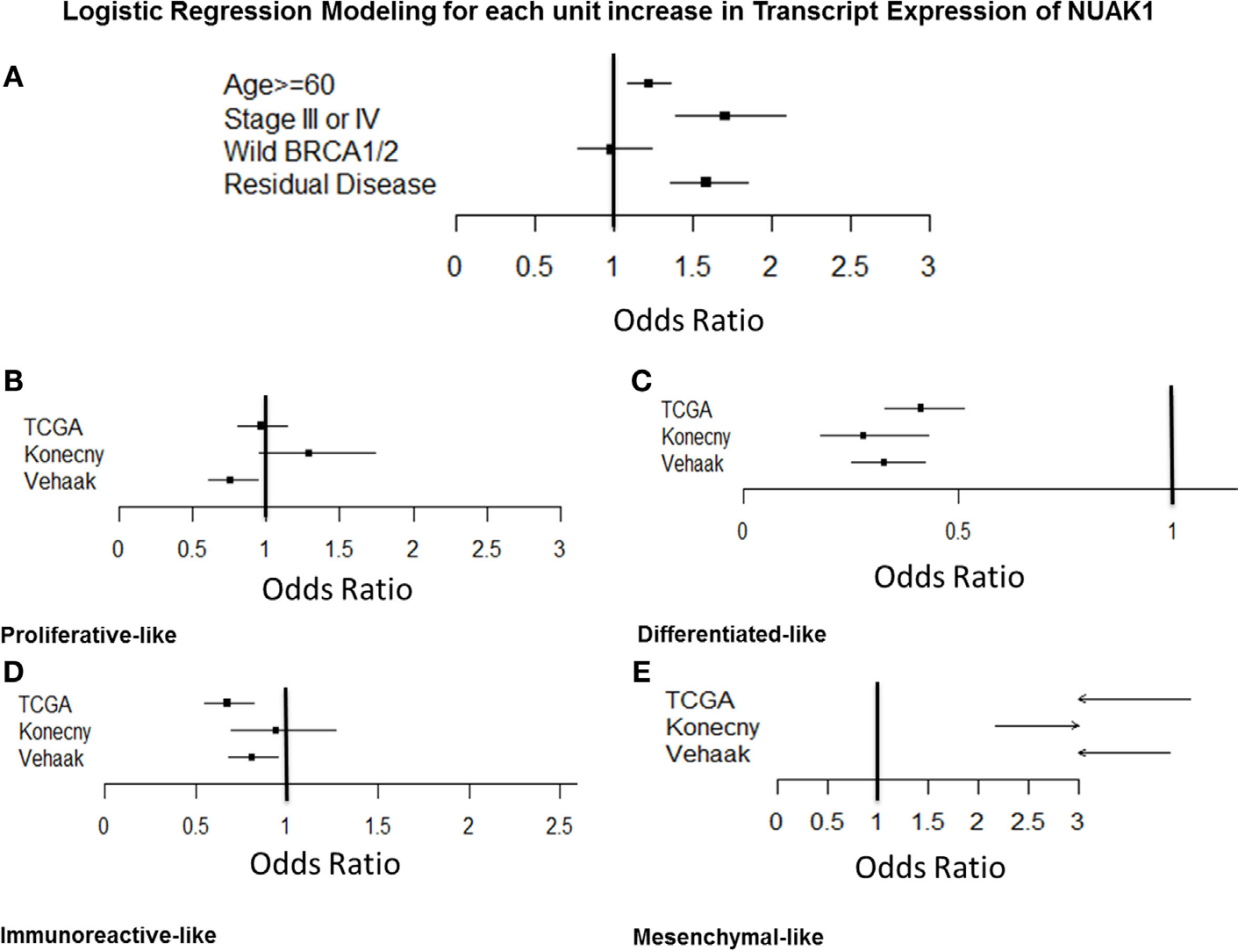
**Odds ratios analyses of NUAK1 transcript expression relative to relevant clinicopathological criteria and molecular disease subtypes of high-grade serous ovarian cancer**. Forest plots detailing odds ratio of patients exhibiting elevated NUAK1 transcript expression relative to clinic-pathological criteria and molecular disease subtypes in public gene expression data. Raw odds ratios results and patient cohorts available for analyses detailed in Table S3 in Supplementary Material.

Logistic regression modeling (Figure 3A; Table S3 in Supplementary Material) demonstrated a relationship between elevated NUAK1 expression and aggressive clinic-pathologic features, as well as select molecular subtypes in ovarian cancer patients, but not with BRCA1/2 status [odds ratio (OR) = 0.93, *p* > 0.05]. Elevated NUAK1 was associated with an increased risk of being diagnosed at an older age, categorized as < vs. ≥60 years old (OR = 1.23, *p* = 0.003) and at advanced stage (OR = 1.73, *p* < 0.0001) as well as with harboring residual disease following primary cytoreductive surgery (OR = 1.58, *p* < 0.0001). TCGA (5), Verhaak et al. (7), and Konecny et al. (8) developed independent molecular classifiers for proliferative, mesenchymal, immunoreactive, and differentiated subtypes of SOC. NUAK1 transcript expression was evaluated in the subset of 472 patients with the TCGA classification, 504 with the Verhaak classification, and 182 with the Konecny classification to determine if NUAK1 varied in any of the molecular subtypes (Figures 3B,E). Elevated expression of NUAK1 was not consistently related to proliferative subtype (Figure 3B) or immunoreactive subtype (Figure 3D) but was consistently less common in patients with the differentiated subtype as defined by all three classifiers with ORs below 0.5 (Figure 3C, *p* < 0.0001) and more common in the TCGA-, Verhaak- and the Konecny-derived mesenchymal subtype with ORs that exceeded 2.0 (Figure 3E, *p* < 0.0001).

### Downregulation of NUAK1 Does Not Sensitize SOC to Cisplatin or Paclitaxel, but Does Attenuate the Migratory Potential of Chemosensitive and Resistant Ovarian Cancer Cells *In Vitro*

To clarify the relationship between elevated NUAK1 and poor PFS, we performed *in vitro* chemosensitization analyses in a model of HGSOC cells (OV90) (25) transfected with a small interfering RNA to silence NUAK1 expression followed by assessment of cisplatin and paclitaxel dose–response (Figure S2A–C in Supplementary Material). These studies revealed that NUAK1 knockdown had no significant impact on relative sensitivity to cisplatin or paclitaxel in OV90 cells.

**Figure 4:**
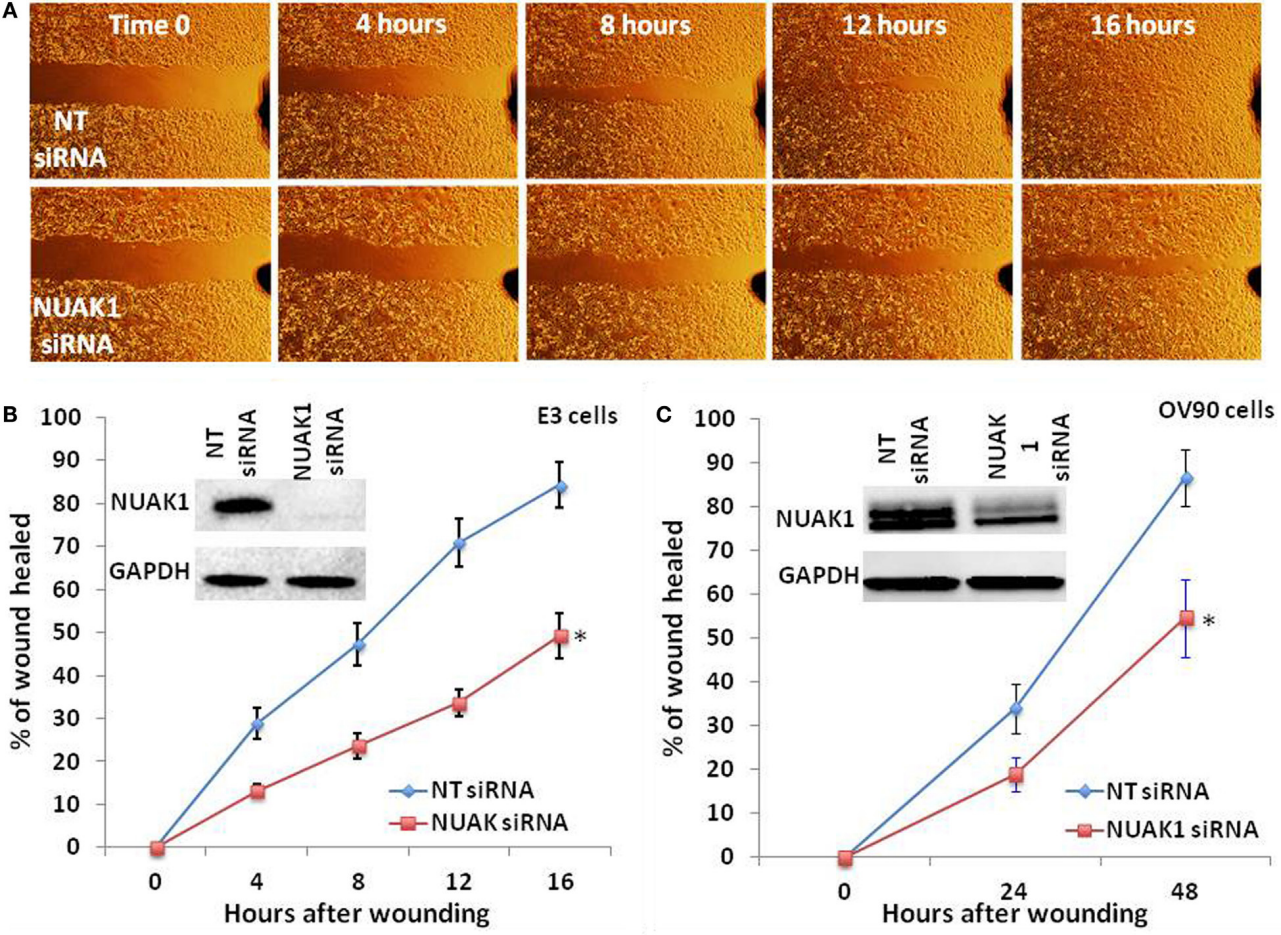
**NUAK1 regulates the migratory potential of chemosensitive and chemoresistant high grade serous ovarian cancer cells**. **(A)** Time-course wound healing assay micrographs of chemoresistant ovarian cancer cells (E3) following NUAK1 knockdown by small interfering RNA (siRNA). **(B)** Representative rates of wound healing in E3 cells following siRNA-mediated knockdown of NUAK1 (*Paired *t*-test *p* = 0.005734). **(C)** Representative rates of wound healing in chemosensitive ovarian cancer cells (OV90) following siRNA-mediated knockdown of NUAK1 (*Paired *t*-test *p* = 0.01315). Inset immunoblot images in **(B,C)** detail confirmation of NUAK1 knockdown. Data in **(B,C)** represent percent of wounds healed relative to areas measured at *t* = 0 h. Data points reflect duplicate, technical replicates, and data sets are representative of triplicate biological replicates.

As we found NUAK1 expression to be associated with both advanced stage and the mesenchymal subtype of HGSOC, we hypothesized that elevated levels of the NUAK1 gene product may increase the migratory potential of HGSOC cells. To test this hypothesis, we silenced NUAK1 expression in OV90 cells (25) and in an in-house generated model of chemorefractory, high-grade HGSOC (E3 cells) (24). Cell lines were transfected with a scrambled control siRNA (non-targeting, NT) or a NUAK1-specific siRNA, and NUAK1 knockdown was confirmed by immunoblot (Figure 4B). Immunoblot analyses revealed a discrete immunoreactive band for NUAK1 in E3 cells (Figure 4B), but a doublet in OV90 cells (Figure 4C), consistent with previous evidence denoting tissue/cell type-specific immunoblot patterns for NUAK1 (26). Wound healing analyses of confluent monolayers of RNAi-transfected ovarian cells revealed significant decreases in migration in NUAK1-silenced cells (Figure 4A (E3 cells); Figures 4B,C). Notably, although the chemoresistant E3 cells were found to be highly migratory relative to OV90 cells (Figures 4B,C, hours after wounding), E3 cells exhibited a greater relative loss of migratory potential following NUAK1 knockdown (Figure 4B) as compared with OV90 cells (Figure 4C).

## Discussion

Nua kinase 1 is a downstream effector of Akt and is activated in response to cellular hypoxia and nutrient starvation (10, 11). Elevated expression of NUAK1 leads to enhanced proliferation, invasion, and metastatic capabilities in diverse tumor cell types (10, 11). Using publicly available transcript expression data from 1,262 ovarian cancer patients, we show that elevated NUAK1 is associated with decreased OS and PFS. We were able to validate the relationship between NUAK1 expression and OS in an independent cohort of 174 ovarian cancer patients with Agilent data. PFS data were, however, not available for the independent cohort with Agilent data. We turned to quantitative PCR in a small cohort of patients with advanced stage < or >2 years PFS to validate our finding that higher transcript expression of NUAK1 was associated with shorter PFS. The association between elevated NUAK1 expression with poor PFS from a publicly available cohort of 802 patients was independently validated by quantitative PCR in an archival tissue set of 25 HGSOC patients with PFS <2 years and 9 HGSOC patients with PFS >2 years, following primary diagnosis. To explore the relationship of NUAK1 and PFS, we assessed whether NUAK1 contributed to chemosensitivity in a model of HGSOC cells (OV90). We silenced NUAK1 gene expression in OV90 cells by RNAi followed by treatment with cisplatin or paclitaxel and found that loss of NUAK1 did not impact chemosensitivity relative to control cells (Figure S2A–C in Supplementary Material).

We, further, observed that elevated NUAK1 is associated with increased odds of harboring residual disease (>R0) and presenting at advanced disease stage. Further, we found that elevated NUAK1 significantly correlates with the mesenchymal disease subtype (5, 7, 8). Previous assessments of molecular ovarian cancer disease subtypes have found that mesenchymal-like disease is associated with a significantly increased risk of poor disease survival (8). Further, we provide functional evidence that NUAK1 regulates tumor cell migration in both chemosensitive (OV90) and chemoresistant (E3) ovarian cancer cell lines. These observations bear directly on the relevance of NUAK1 as a possible therapeutic target in patients that present with chemoresistant or refractory disease. Our comparative analysis of NUAK1 expression levels with relevant clinic-pathologic measures of metastatic spread and patient tumor burden, as well as *in vitro* cell migratory analyses in chemo-sensitive and -resistant models of ovarian cancer cells, confirms previous findings implicating NUAK1 as a key factor supporting invasive and metastatic characteristics of tumor cells.

Overall, our findings corroborate previous evidence that has implicated NUAK1 as a key factor underlying poor disease prognosis in diverse cancer subtypes and expand on recent precedents associating NUAK1 transcript expression with poor outcome in HGSOC patients. Additional studies are required to further the clinical development and deployment of NUAK1 in HGSOC, and to determine the utility of NUAK1 as a potential therapeutic target to mitigate disease progression in HGSOC patients.

## Author Contributions

NP, NB, KC, TL, WO, PT, and JO conducted experiments, interpreted data, and contributed to writing the manuscript; GW analyzed data and contributed to writing the manuscript; GM and CH interpreted data and contributed to writing the manuscript; KD analyzed and interpreted data, and contributed to writing the manuscript; and TC designed and oversaw the project, interpreted data, and contributed to writing the manuscript.

## Disclaimer

The opinions, conclusions, or assertions contained herein are the private views of the authors and are not to be construed as official or as reflecting the views of the Department of the Army, Navy, Air Force, the Department of Defense, or the United States Government.

## Conflict of Interest Statement

The authors declare that the research was conducted in the absence of any commercial or financial relationships that could be construed as a potential conflict of interest.

## Footnotes

^1^http://www.bioconductor.org/.

^2^https://tcga-data.nci.nih.gov/tcga/findArchives.htm or https://tcga-data.nci.nih.gov/tcga/tcgaDownload.jsp.

^3^http://www.ncbi.nlm.nih.gov/geoprofiles.

## Supplementary Material

Table S1 **Univariate and multivariate modeling of NUAK1 transcript expression and overall survival in discovery (*n* = 1262) and validation (*n* = 174) cohorts**.

Table S2 **Univariate and multivariate modeling of NUAK1 transcript expression and overall and progression-free survival in discovery (*n* = 802) cohorts**.

Table S3 **Odds ratios analysis of NUAK1 transcript expression relative to clinic-pathological criteria and molecular disease subtypes**.

Figure S1 **Elevated NUAK1 transcript expression is associated with poor overall survival in serous ovarian cancer patients**. Independent, cross-microarray platform (Agilent) validation. Kaplan–Meier plot of NUAK1 transcript expression relative to overall survival (multivariate continuous HR = 1.029, *p*-value = 0.04, *n* =174).

Figure S2 **NUAK1 does not impact sensitivity to cisplatin or paclitaxel in a cell line model of high grade serous ovarian cancer (OV90 cells)**. **(A)** Immunoblot analyses confirming RNAi-mediated silencing of NUAK1 expression at 96 h after siRNA transfection. **(B)** Cisplatin dose–response analyses of siNUAK1 versus control OV90 cells. **(C)** Paclitaxel dose–response analyses of siNUAK1 versus control OV90 cells. Data reflects triplicate, technical replicates and are representative of two biological replicates.
