# Supplementary material for: NUAK1 (ARK5) Is Associated with Poor Prognosis in Ovarian Cancer
Source: Front Oncol. 2016 Oct 27;6:213. doi: 10.3389/fonc.2016.00213 (PMC5081368; doi:10.3389/fonc.2016.00213)
Supplement: Table S1 — Univariate and multivariate modeling of NUAK1 transcript expression and overall survival in discovery (n = 1262) and validation (n = 174) cohorts. [file Table_1.pdf]

Supplemental Table 1

Cox regression modeling was performed to examine the relationship between NUAK1 transcript expression[ϕ] and overall survival								
Cohort	Univariate Modeling				Multivariate Modeling †			
	N	HR	95% CI	p	N[1]	aHR	95% CI	p
Discovery ¥	1262	1.229	1.147-1.316	4.2E-09	1262	1.19	1.118-1.283	3.03E-07
Validation Ψ	174	2.039	1.094-3.801	0.025	174	1.029	3.46	0.04

N: number of patients, HR (hazard ratio), CI (confidence interval), aHR (stage adjusted hazard ratio).

ϕ NUAK1 transcript expression was evaluated as a continuous variable with HR and 95 CI as well as aHR and 95% CI calculated per unit increase in transcript expression level.

† Multivariate Cox modeling adjusted for stage at diagnosis.

¥ Discovery cohorts with Affymetrix derived transcript data for NUAK1 from Bonome[13], Denkert[14], Dressman[15], Mok[17], Tothill[18], Mateescu[16] and TCGA[3].

Ψ Validation cohort with Agilent derived transcript data for NUAK1 from Konecny[19].
