# Supplementary material for: NUAK1 (ARK5) Is Associated with Poor Prognosis in Ovarian Cancer
Source: Front Oncol. 2016 Oct 27;6:213. doi: 10.3389/fonc.2016.00213 (PMC5081368; doi:10.3389/fonc.2016.00213)
Supplement: Table S2 — Univariate and multivariate modeling of NUAK1 transcript expression and overall and progression-free survival in discovery (n = 802) cohorts. [file Table_2.pdf]

Supplemental Table 2

Cox regression modeling was performed to examine the relationship between NUAK1 transcript expressionᶲ and progression-free survival

Cohort	Univariate Modeling				Multivariate Modeling †			
	N	HR	95% CI	p	N	aHR	95% CI	p
Discovery ¥	802	1.134	1.048-1.227	0.0018	802	1.09	1.006-1.182	0.036

N: number of patients, HR (hazard ratio), CI (confidence interval), aHR (stage adjusted hazard ratio).

ϕ NUAK1 transcript expression was evaluated as a continuous variable with HR and 95 CI as well as aHR and 95% CI calculated per unit increase in transcript expression level.

† Multivariate Cox modeling adjusted for stage at diagnosis.

¥ Discovery cohorts with Affymetrix derived transcript data for NUAK1 from Bonome[13], Denkert[14], Dressman[15], Mok[17], Tothill[18], Mateescu[16] and TCGA[3]. Progression-free survival data was not available for the Validation cohort (Konecny[19]).
