# Supplementary material for: NUAK1 (ARK5) Is Associated with Poor Prognosis in Ovarian Cancer
Source: Front Oncol. 2016 Oct 27;6:213. doi: 10.3389/fonc.2016.00213 (PMC5081368; doi:10.3389/fonc.2016.00213)
Supplement: Table S3 — Odds ratios analysis of NUAK1 transcript expression relative to clinic-pathological criteria and molecular disease subtypes. [file Table_3.pdf]

Supplemental Table 3

Logistic regression modeling was performed using transcript expression of NUAK1 as a continuous variable in the subset of cases with evaluable data to predict advanced age at diagnosis, advanced stage, wildtype BRCA1/2 status, any residual disease or one of the molecular subtypes.

Predicted State		N	OR	95% CI	p-value
Advanced Age at Diagnosis (≥60)		943	1.220	1.091-1.364	0.0005
Advanced Stage (III or IV)		1262	1.703	1.388-2.090	<0.0001
Wildtype BRCA1/2 Status		545	0.979	0.776-1.235	NS
Any Residual Disease		761	1.582	1.354-1.849	<0.0001
Mesenchymal Subtype	– TCGA classifier	472	14.493	8.428-24.923	<0.0001
	– Verhaak classifier	504	5.481	4.020-7.472	<0.0001
	– Konecny classifier	182	3.413	2.213-5.264	<0.0001
Differentiated Subtype	– TCGA classifier	472	0.398	0.316-0.501	<0.0001
	– Verhaak classifier	504	0.317	0.243-0.414	<0.0001
	– Konecny classifier	182	0.265	0.169-0.417	<0.0001
Proliferative Subtype	– TCGA classifier	472	0.957	0.798-1.148	NS
	– Verhaak classifier	504	0.749	0.596-0.941	0.013
	– Konecny classifier	182	1.293	0.944-1.769	NS
Immunoreactive Subtype	– TCGA classifier	472	0.657	0.533-0.811	<0.0001
	– Verhaak classifier	504	0.802	0.675-0.953	0.012
	– Konecny classifier	182	0.946	0.694-1.289	NS

N (number of patients), OR (odds ratio), CI (confidence interval), NS (not significant with p>0.05)
