# Supplementary figures and images for: NUAK1 (ARK5) Is Associated with Poor Prognosis in Ovarian Cancer
Source: Front Oncol. 2016 Oct 27;6:213. doi: 10.3389/fonc.2016.00213 (PMC5081368; doi:10.3389/fonc.2016.00213)

Supplemental Figure 1

Overall Survival

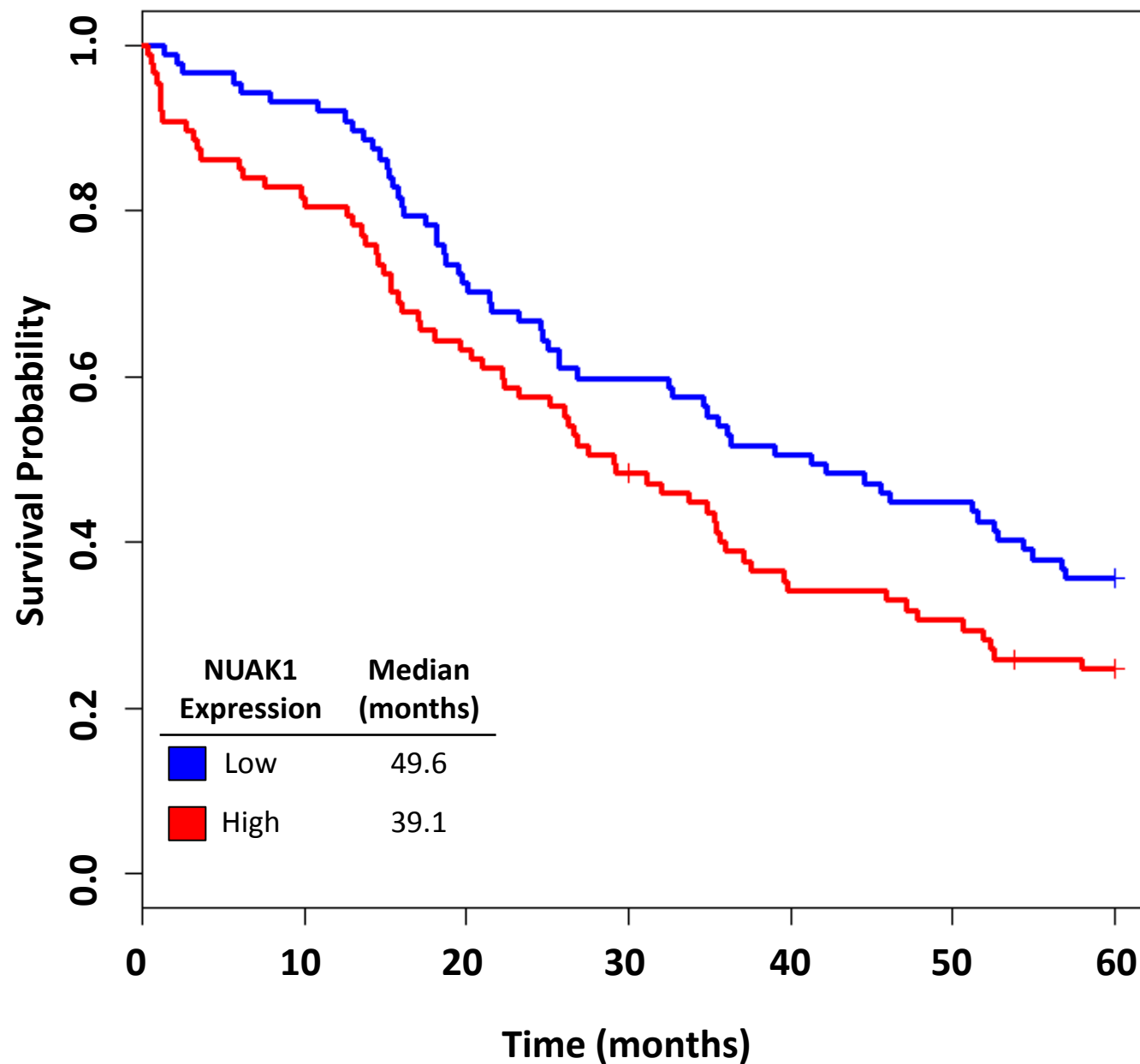

Supplement: Figure S1 — Elevated NUAK1 transcript expression is associated with poor overall survival in serous ovarian cancer patients. Independent, cross-microarray platform (Agilent) validation. Kaplan–Meier plot of NUAK1 transcript expression relative to overall survival (multivariate continuous HR = 1.029, p-value = 0.04, n =174). [file Image_1.pdf]

Supplemental Figure 2

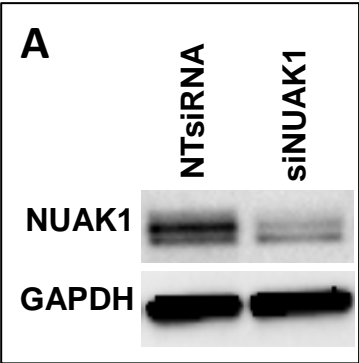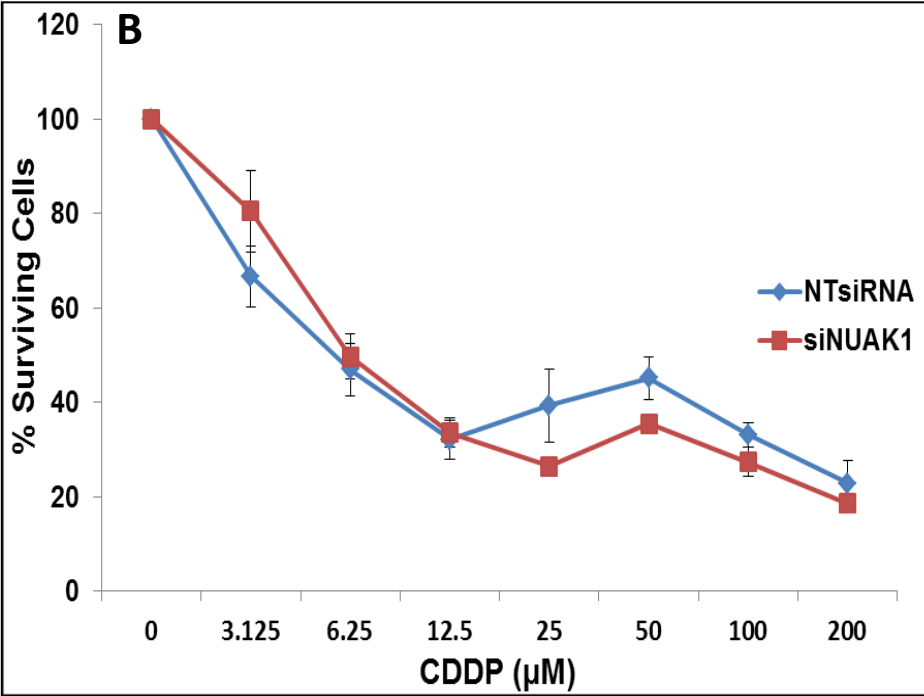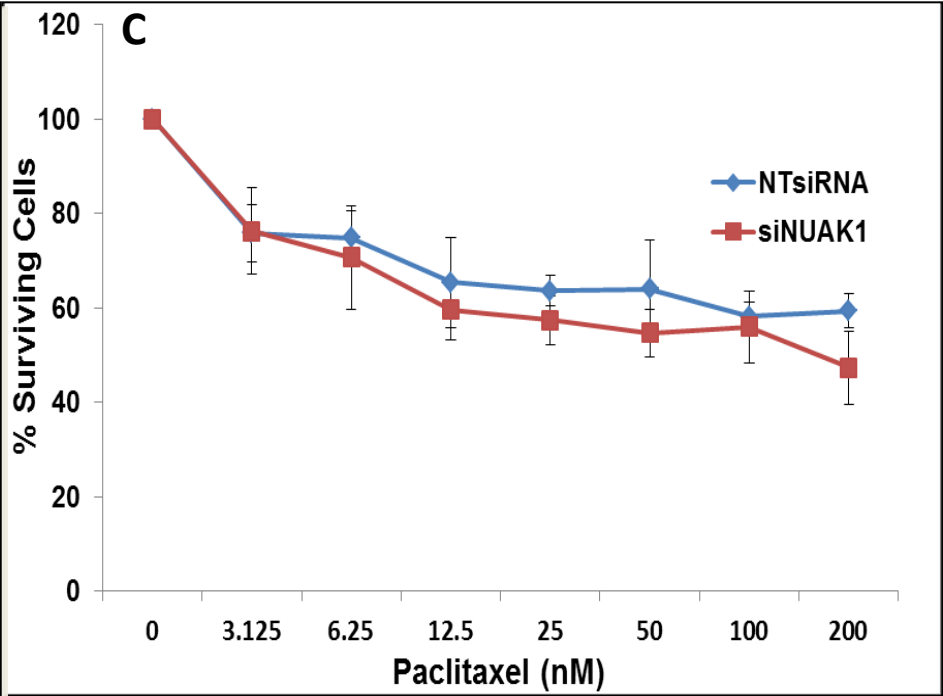

Supplement: Figure S2 — NUAK1 does not impact sensitivity to cisplatin or paclitaxel in a cell line model of high grade serous ovarian cancer (OV90 cells). (A) Immunoblot analyses confirming RNAi-mediated silencing of NUAK1 expression at 96 h after siRNA transfection. (B) Cisplatin dose–response analyses of siNUAK1 versus control OV90 cells. (C) Paclitaxel dose–response analyses of siNUAK1 versus control OV90 cells. Data reflects triplicate, technical replicates and are representative of two biological replicates. [file Image_2.pdf]
